# Localized DNA Demethylation at Recombination Intermediates during Immunoglobulin Heavy Chain Gene Assembly

**DOI:** 10.1371/journal.pbio.1001475

**Published:** 2013-01-29

**Authors:** Roza Selimyan, Rachel M. Gerstein, Irina Ivanova, Patricia Precht, Ramesh Subrahmanyam, Thomas Perlot, Frederick W. Alt, Ranjan Sen

**Affiliations:** 1 Laboratory of Molecular Biology and Immunology, National Institute on Aging, National Institutes of Health, Baltimore, Maryland, United States of America; 2 Department of Molecular Genetics and Microbiology, University of Massachusetts Medical School, Worcester, Massachusetts, United States of America; 3 The Howard Hughes Medical Institute, The Children's Hospital, Immune Disease Institute and Department of Genetics, Harvard Medical School, Boston, Massachusetts, United States of America; Scripps Research Institute, United States of America

## Abstract

Multiple epigenetic marks have been proposed to contribute to the regulation of antigen receptor gene assembly via V(D)J recombination. Here we provide a comprehensive view of DNA methylation at the immunoglobulin heavy chain (IgH) gene locus prior to and during V(D)J recombination. DNA methylation did not correlate with the histone modification state on unrearranged alleles, indicating that these epigenetic marks were regulated independently. Instead, pockets of tissue-specific demethylation were restricted to DNase I hypersensitive sites within this locus. Though unrearranged diversity (D_H_) and joining (J_H_) gene segments were methylated, DJ_H_ junctions created after the first recombination step were largely demethylated in pro-, pre-, and mature B cells. Junctional demethylation was highly localized, B-lineage-specific, and required an intact tissue-specific enhancer, Eμ. We propose that demethylation occurs after the first recombination step and may mark the junction for secondary recombination.

## Abstract 2

The dynamics of DNA methylation during the complex genomic rearrangement of antigen receptor genes in developing B lymphocytes reveal localized demethylation of the first recombination product that may serve as a mark necessary for the second step of rearrangement.

## Author Summary

DNA methylation at CpG dinucleotides is implicated in the regulation of gene expression in mammals. However, the regulation of DNA methylation itself is less clear despite recent advances in identifying enzymes that add or remove methyl groups. We have investigated the dynamics of DNA methylation during genome rearrangements that assemble antigen receptor genes in developing B lymphocytes to determine whether methylation status correlates with rearrangement potential. Two recombination events generate immunoglobulin heavy chain (IgH) genes. The first step brings together diversity (D_H_) and joining (J_H_) gene segments to produce DJ_H_ junctions. We show that both gene segments are methylated prior to rearrangement, whereas the DJ_H_ product is demethylated. DJ_H_ junctional demethylation is tissue-specific and requires an enhancer, Eμ, located within the IgH locus. The latter observations indicate that localized demethylation of the DJ_H_ junction occurs after the first recombination step and thus does not guide this first step of IgH gene assembly. Our working hypothesis is that recombination induces demethylation of recombinant product and may mark the junction for the second step of IgH rearrangement, juxtaposition of variable (V_H_) gene segments to rearranged DJ_H_ products to produce fully recombined V(D)J alleles.

## Introduction

Tissue-specific gene expression requires multiple epigenetic changes. These include nuclear location, chromatin remodeling, covalent histone modifications, and DNA methylation [1]–[4]. Recent genome-wide analyses reveal several important correlations between gene activity and epigenetic modifications. Transcriptionally inactive genes are marked by histone H3 methylation at lysines 9 and 27 (H3K9me, H3K27me), whereas histone acetylation and H3K4 methylation are associated with gene activity [5]. H3K9 acetylation and H3K4 methylation have been inversely correlated with CpG methylation, consistent with the long-established view that transcriptionally active promoters are hypomethylated [6].

Most studies of CpG methylation vis-à-vis transcription have focused on gene promoters, particularly those that contain CpG islands (CGI). Such promoters are typically un-methylated and are transcriptionally active; conversely, methylated CGI promoters are usually transcriptionally silent. The role of CpG methylation in non-CGI promoters is less clear [7]. Many tissue-specific promoters fall in this category and, in a limited number of cases where examined, such promoters also have reduced CpG methylation in tissues where they are active. Most recently, the advent of whole genome CpG mapping has drawn attention to possible functions of CpG methylation within gene bodies [8],[9]. Proposed functions for such CpGs include transcription elongation and the regulation of splicing. The lack of a coherent picture for the role of CpG methylation in non-CGI contexts indicates an ongoing need for analysis of CpG methylation, particularly in tissue-specific genes that lack CGIs.

**Figure 1:**
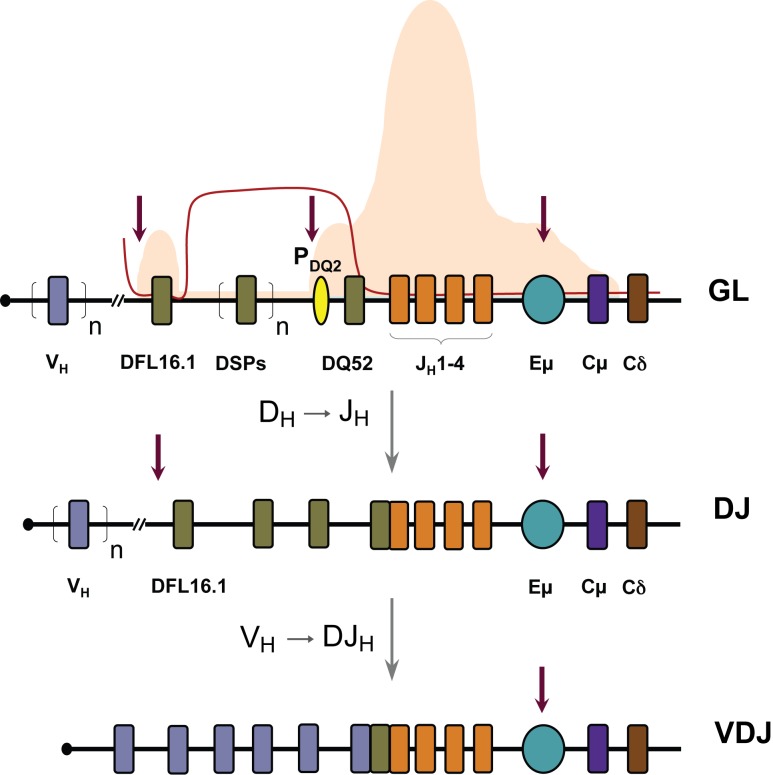
Immunoglobulin heavy chain locus and B cell development. Schematic of the murine IgH locus showing variable (V_H_, blue, n represents approximately 150 V_H_ gene segments), diversity (D_H_, grey, n represents six to nine DSP gene segments), and joining (J_H_, orange) gene segment [11],[12]. Exons encoding the constant regions of IgM and IgD are indicated as Cμ and Cδ. A promoter 5′ of DQ52, the 3′-most D_H_ gene segment, is indicated by the yellow oval and the intronic enhancer Eμ by a teal oval. Top line shows the germline (GL) configuration with associated histone modifications in B lineage precursors [23]–[25]. Histone H3 and H4 acetylation are shown in orange and presence of heterochromatic H3K9 methylation by the red line. Vertical red arrows represent the tissue-specific DNase I hypersensitive sites in the germline state. Next two lines show sequential stages of VDJ recombination at the IgH locus. D_H_ to J_H_ rearrangement occurs first resulting in a DJ_H_ junction and, depending on which D_H_ rearranges, residual upstream unrearranged D_H_s may be present. V_H_ rearrangement occurs to the DJ_H_ junction to generate a VDJ_H_ junction; during this process unrearranged D_H_s are lost from the genome.

Antigen receptor genes of B and T lymphocytes serve as excellent paradigms for developmentally regulated gene expression. Immunoglobulin heavy chain (IgH) genes are assembled during B lymphocyte development by juxtaposition of variable (V_H_), diversity (D_H_), and joining (J_H_) gene segments by a process known as V(D)J recombination [10]. The mouse genome contains approximately 150 V_H_ gene segments, 10 to 13 D_H_ gene segments, and four J_H_ gene segments, dispersed over 2 Mb [11],[12]. D_H_ to J_H_ recombination occurs first, followed by V_H_ recombination to the rearranged DJ_H_ junction (Figure 1). These two steps produce a fully recombined V(D)J allele, with the potential to encode heavy chain protein. IgH expressing cells develop into pre-B cells where immunoglobulin κ or λ light chain genes rearrange. Cells that make both IgH and IgL polypeptides express cell surface immunoglobulin, and are exported out of the bone marrow to become mature functional B cells.

DNA methylation was the earliest epigenetic mark associated with immunoglobulin gene regulation [13],[14]. Using a variety of cell lines, these pioneering studies showed that Vκ promoters that were rearranged, and therefore transcriptionally active, lacked CpG methylation. Because these studies used restriction enzyme isoschisomers, it was difficult to define the extent of demethylation on recombined κ alleles in these studies; however, a site located approximately 10 kb 5′ of the promoter of a rearranged Vκ21 remained methylated. Subsequently, studies by Bergman, Cedar and colleagues showed that one κ allele is preferentially demethylated at a site close to the Cκ exons at the pre-B cell stage of B cell differentiation. Preferential Vκ recombination of the demethylated allele led them to propose that demethylation permits Vκ to Jκ recombination [15]. Analyses of transgenic mice containing synthetic recombination substitutes were also consistent with the idea that demethylated alleles recombine preferentially [16],. Additionally, de-methylation state of this site has also been proposed to play a role in somatic hypermutation in germinal centers [18].

Compared to these extensive studies at the κ locus, relatively little is known about developmental regulation of DNA methylation at the IgH locus. Two relatively recent studies evaluated the role of methylation in V_H_ gene rearrangements [19],[20]. Within the small family of V_H_S107 genes, the methylation status of one CpG in pro-B cells correlated with recombination potential. Both studies also examined the status of the 3′-most V_H_ gene segment, V_H_ 81X, that rearranges most frequently. Two CpG sites within the coding region remained methylated in pro-B cells whereas a CpG near the recombination signal sequence was demethylated. However, the extent of demethylation of all genes examined was comparable in pro-B cells where V_H_ genes recombine and in non-B lineage cells where V_H_ genes do not recombine. Thus, methylation status could not be directly correlated with rearrangement potential. More importantly, virtually nothing is known about CpG methylation in D_H_–Cμ part of the locus, where V(D)J recombination is initiated [21].

Here we used the bisulfite modification method to assay DNA methylation in the D_H_–Cμ domain of the IgH locus prior to the onset of recombination and as recombination proceeds. The only sites of extensive tissue-specific DNA demethylation in the unrearranged locus corresponded to a promoter close to DQ52 and the intronic enhancer Eμ. Additionally, sequences close to the intergenic control region [22], which marks the 5′ boundary of the D_H_-Cμ domain, were partially demethylated. These regions correspond to the only known DNase1 hypersensitive sites in this part of the IgH locus. All CpGs close to, or within, other re-arrangeable D_H_ or J_H_ gene segments remained hypermethylated regardless of whether they were marked with activation or inactivation histone modifications. However, DJ_H_ junctions that were generated after the first recombination step were completely demethylated. Junctional demethylation was highly localized, B lineage-specific, and required the presence of an intact Eμ. These observations suggest that localized demethylation of the DJ_H_ junction occurs after the first recombination step and may mark the junction for subsequent V_H_ gene rearrangements.

## Results

### Tissue-Specific DNA Demethylation Coincides with DNase I Hypersensitive Sites

Histone modifications across the unrearranged D_H_–Cμ domain of the IgH locus show marked heterogeneity. The region encompassing the J_H_ gene segments is marked by the highest levels of histone acetylation and histone H3 trimethylation at lysine 4 (H3K4me3) (Figure 1) [23]–[25]. The 5′- and 3′ D_H_ gene segments, DFL16.1 and DQ52, are also associated with these active modifications, whereas the six to nine DSP gene segments that lie in between are marked by repressive H3 dimethylation at lysine 9 (H3K9me2). Two tissue-specific DNase l hypersensitive sites (DHS) in this part of the locus correspond to a promoter associated with DQ52 and the intronic enhancer Eμ. A cluster of three DHSs was recently identified just 5′ of DFL16.1 [22]. These latter regions contain binding sites for the transcription factor CTCF [26] and have been proposed to serve as the 5′ boundary of the D_H_–Cμ domain [27].

To investigate the DNA methylation state of the unrearranged locus, we purified genomic DNA from pro-B cells obtained from the bone marrow of RAG2-deficient mice to use in bisulfite modification assays modified from Frommer et al. [28]. We used at least two independent DNA preparations starting with cells obtained from six mice for each sample. Methylation profiles of the regions amplified from two independent experiments were comparable (Figure S1). We sequenced approximately 12–50 alleles (Table S1), of which 12 representative clones are shown throughout the text. The mb-1 promoter and β-globin locus control region served as positive [29] and negative controls [30], respectively (Figure S2); we used CD4^+^ CD8^+^ double positive thymocytes (DP) as a lineage control and kidney genomic DNA as a non-lymphoid control. The relative positions of all CpGs are shown in Figure S3.

**Figure 2:**
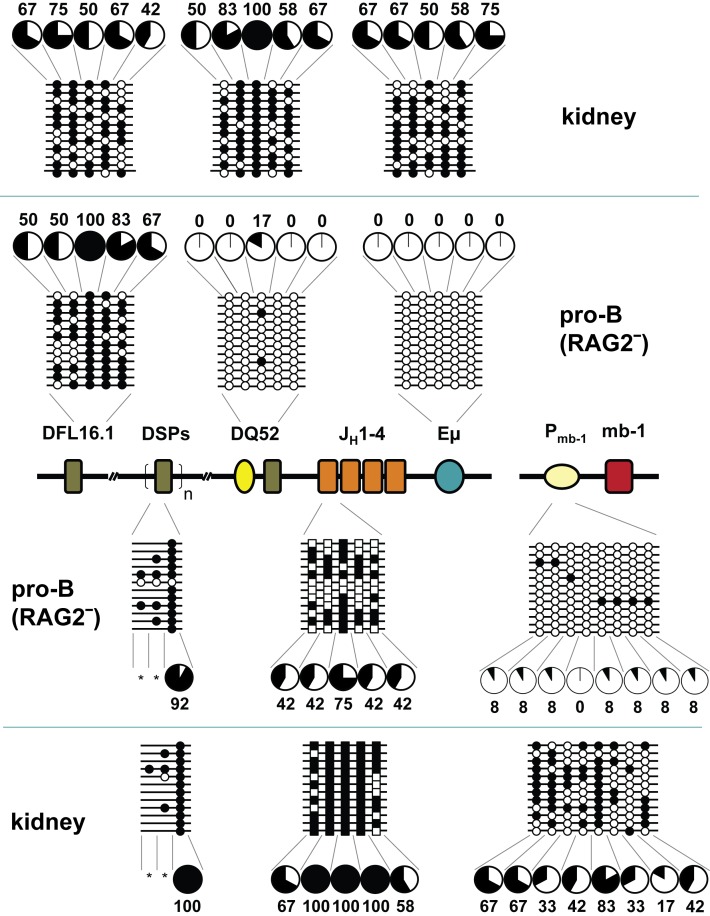
DNA methylation in the D_H_-Cμ domain of the IgH locus. Purified genomic DNA from CD19^+^ pro-B cells obtained from the bone marrow of RAG2-deficient mice was used in bisulfite modification assays. 400–500-bp amplicons corresponding to D_H_ and J_H_ gene segments and regulatory sequences (DQ52 and Eμ) as indicated were cloned and sequenced. Each horizontal line represents the methylation status of a sequenced allele and each vertical line methylation of a specific cytosine residue. J_H_1-associated CpGs are indicated as squares. Filled and open circles, or squares, indicate methylated and unmethylated cytosines, respectively. Methylation at each position is summarized in the form of pie charts with the percentage of methylated residues indicated numerically. Asterisks replace pie charts where the number of sequences falls below 12. Data shown are derived from at least two independent preparations of pooled bone marrow cells obtained from four to six mice each. Kidney genomic DNA served as the non-B lineage control and the previously characterized mb-1 promoter served as a B lineage-specific positive control. Primers for the DSP region amplify all DSP gene segments from the repeat array. Because the numbers of CpGs vary between DSP gene segments, three different CpG patterns are evident in this amplicon.

We found that sequences near DQ52 and Eμ were substantially demethylated in RAG2-deficient pro-B cell DNA compared to kidney DNA (Figure 2). In contrast, unrearranged DFL16.1 and DSP gene segments, which are marked by active and inactive histone modifications [23], respectively, were methylated. While the methylation status of DSP gene segments was consistent with earlier association of H3K9me2 modification with CpG methylation [31], the methylated state of DFL16.1 did not follow this paradigm. An amplicon that included the J_H_1 gene segment located within the peak of active modifications was partially demethylated in pro-B cells compared to kidney. Note that the DQ52 and J_H_1 amplicons, that are demethylated and partially methylated, respectively, are separated by only about 170 nucleotides that contain two CpGs. These observations indicate that the prominent peak of H3ac and H3K4me3 encompassing the J_H_ gene segments is not sufficient to direct CpG demethylation of this region. Moreover, the J_H_ region has been shown to bind the highest levels of recombinase proteins RAG1 and 2, to form a recombination center at which IgH rearrangement initiates [21]. Clearly, the partially methylated state of this region does not preclude RAG 1/2 recruitment. Additionally, DQ52 and DFL16.1 have been shown to recombine most frequently among D_H_ gene segments [32]–[35], yet they had distinctly different methylation states. The methylation status of D_H_ and J_H_ gene segments suggests that the first step of Ig gene assembly may not be regulated by DNA methylation.

**Figure 3:**
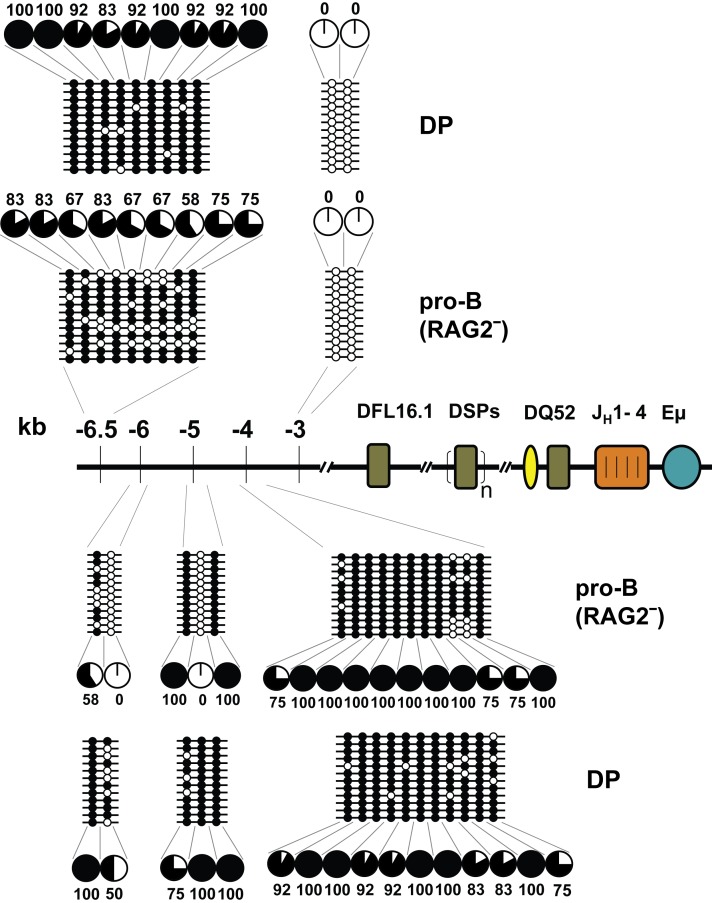
DNA methylation status of DNase 1 hypersensitive sites 5′ of DFL16.1. Three newly identified DNase 1 hypersensitive sites are located at approximately 6–6.5, 4–4.5, and 0.4–1.3 kb 5′ of DFL16.1. Genomic DNA from primary RAG-deficient pro-B cells and DP (CD4^+^ CD8^+^) thymocytes were used in bisulfite mapping experiments to examine CpG methylation. Five amplicons covering regions between 3 kb and 7 kb 5′ of DFL16.1 were modified, cloned, and sequenced. The distribution of CpG dinucleotides within each amplicon are noted in Figure S3. Filled and open circles represent methylated and unmethylated residues, respectively. Pie charts summarize the percentage of methylated alleles at each position.

Because both DQ52 and Eμ coincide with tissue-specific DHSs, we examined the methylation status of newly identified DHSs located approximately 6, 4, and 1 kb 5′ of DFL16.1. We found two clusters of CpG dinucleotides centered approximately at 6.5 and 4 kb 5′ of DFL16.1. The precise location of these regions and the relative location of CpG dinucleotides within each region are shown in Figure S3. The cluster at −6.5 was partially demethylated in primary pro-B cells compared to DP thymocytes that served as a non-B lineage control (Figure 3). The cluster at −4 kb was similarly methylated in both cell types. Because neither location corresponded precisely with the DHSs, we also examined shorter stretches of CpG dinucleotides in this region. Two sites located at −6 kb were significantly demethylated in pro-B cells compared to DP thymocytes, whereas one out of three CpGs at −5 kb was completely demethylated in pro-B cells. Two CpGs at −3 kb were completely demethylated in both pro-B cells and DP thymocytes. Finally, a cluster of CpGs in the −1.3 kb region was largely methylated in pro-B cells (see below). Overall, the majority of sites in this region remained methylated in pro-B cells, with very specific sites being targeted for demethylation. Possible interpretations of these observations are discussed below.

### Localized DNA Methylation at Recombination Intermediates

**Figure 4:**
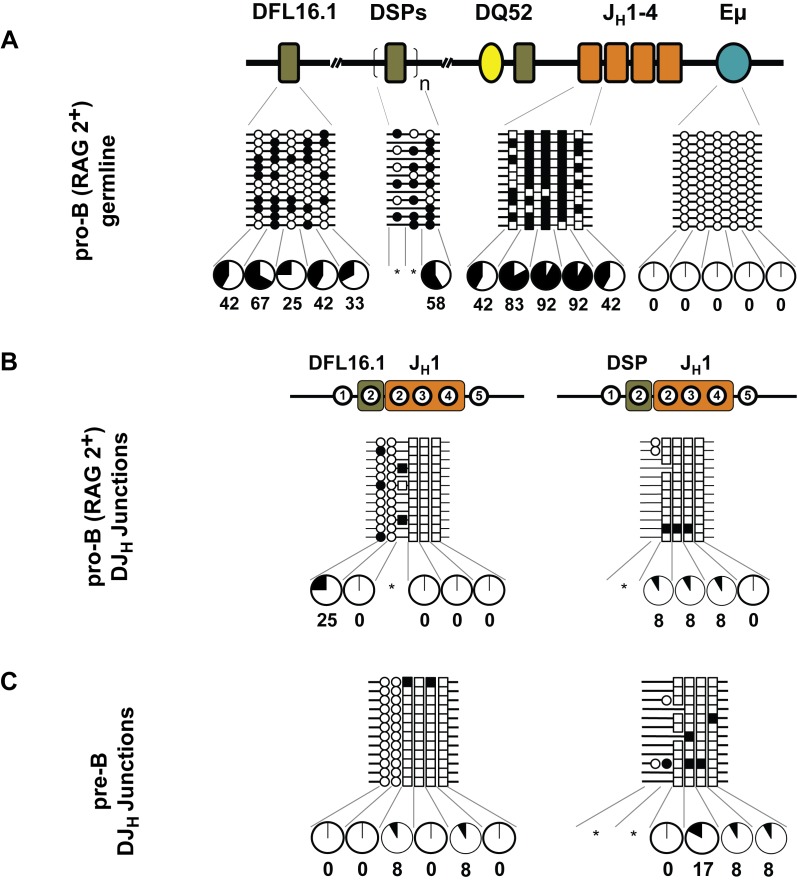
DNA methylation status after the first step of IgH locus recombination. Pro-B cells were purified from the bone marrow of wild-type C57BL/6 mice. Data shown are derived from two independent preparations of pro- and pre-B cells obtained from six to eight mice in each experiment. This cell population contains a mix of germline and partially rearranged IgH alleles. After bisulfite modification, the genomic DNA was used to amplify unrearranged (A) and DJ_H_ rearranged junctions containing DFL16.1 and DSP gene segments (B). Cytosines derived from D_H_ and J_H_ gene segments are marked as circles and squares, respectively. Filled and open circles, or squares, indicate methylated and unmethylated cytosines, respectively. Numbers within regions marked as DFL16.1, DSP and J_H_1 in (B) denote CpG dinucleotides corresponding to the configuration of these residues at the respective unrearranged gene segments. For example, of the five CpGs at unrearranged DFL16.1 only the first two are retained in DFL16.1/J_H_1 junctions. The total numbers of CpG dinucleotides are reduced in junctional sequences because some residues are lost during VDJ recombination as described in the text. Additional heterogeneity is due to the imprecise nature of recombination. Pie charts summarize the percentage of methylated cytosines at each position, except where the number of alleles falls below 12 (indicated by asterisks). (C) Methylation state of recombined DJ_H_ alleles in purified pre-B cells. This population contains a mix of VDJ_H_ recombined and DJ_H_ recombined IgH alleles (see Figure 5A). Note that the number of circles and squares representing D_H_- or J_H_-associated CpGs differed in the DJ_H_ junctions compared to the corresponding unrearranged regions due to junctional variability.

To determine whether DNA methylation was altered by recombination, we examined pro-B cells purified from the bone marrow of wild-type mice, which contain DJ_H_ junctions as well as unrearranged alleles. We found that unrearranged DFL16.1, DSP, and J_H_ regions were methylated, while Eμ region was demethylated (Figure 4A) as seen in RAG2-deficient pro-B cells (note that un-rearranged DFL16.1 and DSP regions in these cells represent fully germline IgH alleles as well as those in which a downstream D_H_ gene segment has undergone recombination to form a DJ_H_ junction). However, DFL16.1/J_H_1 and DSP/J_H_1 recombined junctions were demethylated (Figures 4B and S4). For junctions that involved DFL16.1, demethylation of both parts of the recombination reaction was evident due to the presence of analyzable CpGs in each segment. Many DSP-involving junctions lacked CpG dinucleotides contributed from the DSP part. This is because all DSP gene segments lie within a 4-kb repeat unit [23],[36], which is reflected in all DSP family members sharing a common CpG located 3′ of the gene segment. Two DSP gene segments, DSP2.2 and DSP2.3, have a CpG within the gene segment. In addition, the amplicon encompassing DSP2.3, includes a third CpG dinucleotide 5′ of the gene segment (Figure S3). The 3′ CpG is lost during recombination, and deletions that occur during recombination lead to many junctions that have no CpG contribution from the D_H_ part (Figures 4B, 4C, and S4). Yet, demethylation of DSP/J_H_ junctions was evident from the state of residual CpGs that fell within the J_H_1 region.

During B cell development, pro-B cells that contain a functional IgH allele undergo several rounds of cell division and differentiate into small pre-B cells [37]. We found that pre-B cells also contained demethylated DFL16.1/J_H_1 and DSP/J_H_1 junctions (Figure 4C). Sequence diversity at recombination junctions provided an unequivocal measure of allelic individuality in our experiments. For example, many completely demethylated alleles, such as DFL16.1/J_H_1 junctions in pre-B cells, could have arisen by aberrant amplification of a single allele. However, analysis of the junctional sequences (Figure S4) showed that each of these represented unique alleles.

**Figure 5:**
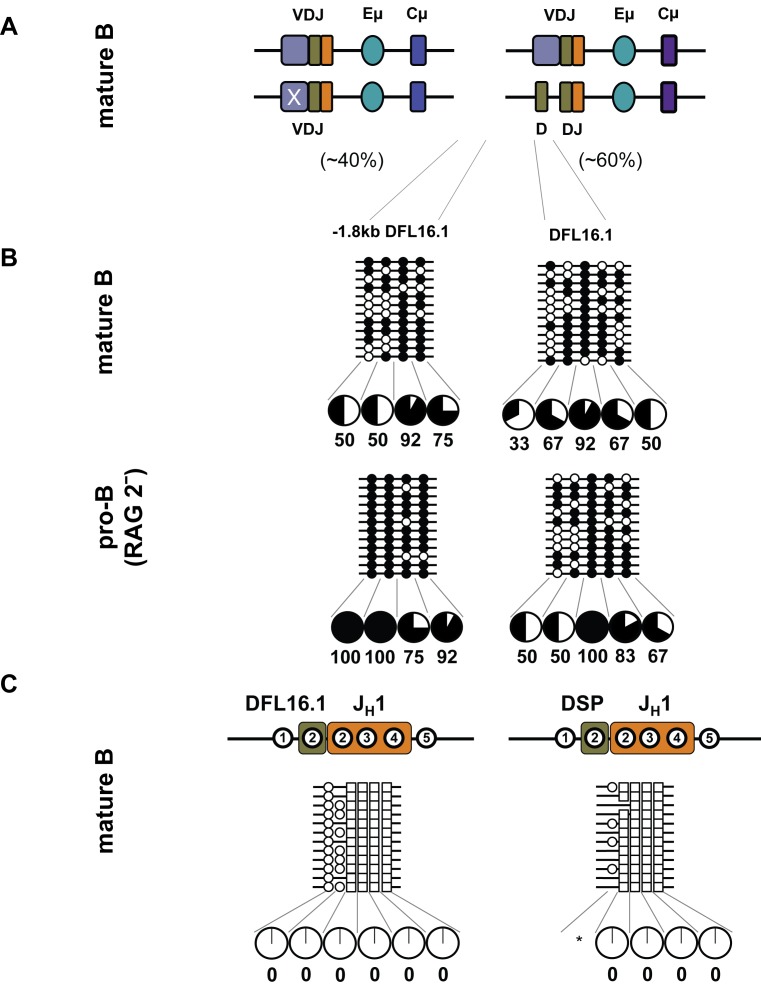
DNA methylation state of unrearranged and DJ_H_ recombined alleles in mature B cells. (A) Mature B cells were purified from spleens of C57BL/6 mice, and the genomic DNA was subjected to bisulfite modification assays. 40% of these cells contain two VDJ_H_ recombined alleles and the remainder contains one VDJ_H_ and one DJ_H_ recombined allele. (B) Amplicons corresponding to unrearranged DFL16.1 gene segment and a region centered 1.3 kb 5′ to DFL16.1 were cloned and sequenced. For comparison, methylation of the same region in pro-B cells derived from RAG2-decificient bone marrow is shown in the bottom panel. Filled and open circles indicate methylated and unmethylated cytosines. Pie charts summarize the percentage of methylated cytosines at each position; data are derived from two independent spleen B cell preparations with two to four mice in each experiment. (C) DJ_H_ junctions were amplified from bisulfite modified DNA, followed by cloning and sequencing. Circles and squares represent cytosines from D_H_ and J_H_1 gene segment, respectively. Filled and open circles, or squares, indicate methylated and unmethylated cytosines, respectively. Numbers within regions marked as DFL16.1, DSP, and J_H_1 denote CpG dinucleotides corresponding to the configuration at the respective unrearranged gene segments. For example, of the five CpGs at unrearranged DFL16.1, only the first two are retained in DFL16.1/J_H_1 junctions. Variations in the total number of cytosines are due to imprecise joining during VDJ recombination. Pie charts summarize the percentage of methylated cytosines. The asterisk indicates positions where less than 12 CpGs were observed due to reduced representation caused by junctional diversity.

Demethylated DJ_H_ junctions could arise by preferential juxtaposition of demethylated D_H_ and J_H_ gene segments; alternatively, demethylation could be targeted to the junction after recombination. One prediction of allelic demethylation prior to recombination is that unrearranged D_H_s located 5′ of the DJ_H_ junction (see Figure 1) should be demethylated. To determine whether DNA demethylation was specific to the DJ_H_ junction, or occurred more widely over recombinant allele, we used mature B cells. Earlier studies have shown that about 40% of mature B cells from wild-type mice contain VDJ_H_ rearrangement on both alleles [37],[38]. In such cells, unrearranged D_H_ gene segments are lost from the genome. The remainder contain one VDJ_H_ rearranged allele and one DJ_H_ rearranged allele; these cells therefore contain unrearranged D_H_ gene segments 5′ to the rearranged DJ_H_ junction (Figure 5A). Mature B cells with germline IgH alleles have not been identified in normal mice. First, we determined the methylation state of the DJ_H_ junctions in mature B cells. As seen in pro- or pre- B cells, DFL16.1/J_H_1 and DSP/J_H_1 junctions were demethylated in mature B cells (Figure 5C). We then analyzed germline DFL16.1 in mature B cells to score for all alleles with downstream D_H_ rearrangements. We also analyzed a region approximately 1.3 kb 5′ of DFL16.1 that would be intact on DFL16.1 rearranged alleles. We found that both these regions were hypermethylated in mature B cells approximating that seen in RAG2-deficient pro-B cells (Figure 5B). We conclude that demethylation of recombined alleles is highly localized to the DJ_H_ junction.

### DNA Demethylation Requires the Intronic Enhancer Eμ

To understand the basis for localized demethylation, we considered the following possibilities. First, a specific D_H_ gene segment and the J_H_ region could be demethylated immediately before recombination. In this model demethylation would precede rearrangement and could possibly target the recombinase to a pre-selected D_H_ gene segment. Second, demethylation may occur during recombination, after the DNA breaks have been introduced by recombinase. Third, demethylation of the DJ_H_ junction could occur after rearrangement. The last model predicts the existence of methylated DJ_H_ junctions that are somehow trapped between recombination and demethylation steps. As described below, we found two sources of such DJ_H_ recombined alleles.

**Figure 6:**
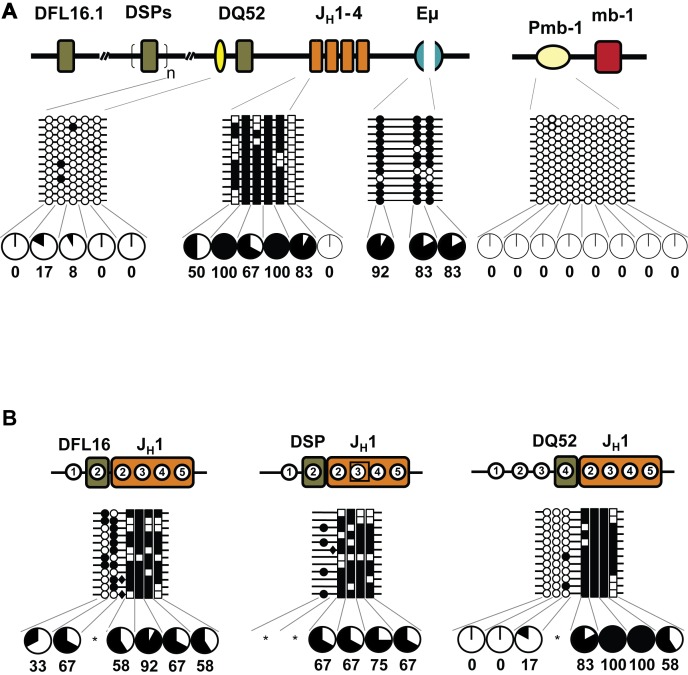
DNA methylation state of IgH alleles in the absence of the intronic enhancer Eμ. Pro-B cells were purified from the bone marrow of Eμ-deficient mice [40]. Genomic DNA was subjected to bisulfite modification followed by PCR amplification of germline gene segments (A) and DJ_H_ junctions (B). The 220-bp deletion of Eμ is represented as partial blue arcs separated by a gap. Filled and open circles, or squares, indicate methylated and unmethylated cytosines, respectively. Numbers within regions marked as DFL16.1, DSP, and J_H_1 denote CpG dinucleotides corresponding to the configuration at the respective unrearranged gene segments. For example, of the five CpGs at unrearranged DFL16.1 only the first two are retained in DFL16.1/J_H_1 junctions. Three out of five cytosines used to analyze wild-type alleles remain in this deletion, and their methylation status is shown immediately below the disrupted enhancer. Sequences that substitute for the enhancer also contain CpGs whose methylation status is shown in Figure S5. (B) Recombined DFL16.1, DSP and DQ52 to J_H_1 junctions were amplified, cloned, and sequenced. Untemplated CpGs incorporated during VDJ recombination were found to be methylated (filled diamonds). Data shown are derived from two independent preparations of pro-B cells from Eμ-deleted mice, with four to six mice in each experiment.

The intronic enhancer Eμ controls key features of the IgH locus assembly via its effects on chromatin structure. Of note, Eμ-deleted IgH alleles undergo D_H_ recombination but not V_H_ recombination [39],[40]. We have recently found that the Eμ enhancer directs localized histone modification changes at DJ_H_ junctions and proposed that these changes configure the IgH locus for V_H_ gene recombination [41]. To determine whether Eμ affected the methylation status of the DJ_H_ junction, we applied the bisulfite procedure to genomic DNA of bone marrow pro-B cells from mice that lack a 220 nucleotide Eμ core [40]. We first examined the two regulatory regions. Three of five CpG sites used to assess Eμ DNA methylation flank the core enhancer and are therefore present on the deleted alleles. These three sites were highly methylated on Eμ-deficient alleles (Figure 6A). CpGs associated with the juxtaposed loxP sites that substitute the Eμ core were also hypermethylated (Figure S5). Thus, a functional enhancer is required to demethylate this region. The J_H_1 region remained hypermethylated in Eμ^−^ alleles, whereas the DQ52 region was unmethylated on Eμ^−^ alleles indicating that its demethylation was Eμ-independent (Figure 6A).

In striking contrast to our observations on Eμ-sufficient alleles, we found that DFL16.1/J_H_1and DSP/J_H_1 junctions were hypermethylated in Eμ-deficient pro-B cells (compare Figure 6B to Figures 4B, 4C, and 5C). Moreover, non-templated CpGs introduced by N region addition during recombination [37] were also methylated (Figure 6B, indicated by black diamonds). Interestingly, DQ52/J_H_1 junctions were of a hybrid nature, consisting of several demethylated CpGs from DQ52 and several methylated CpGs from the J_H_1 portions of the junction. We infer that demethylation at DJ_H_ junctions requires Eμ. The state of DQ52/J_H_1 junctions indicated that neither rearranging segment imposed its pattern of methylation during the “cut-and-paste” reaction of V(D)J recombination. These observations are consistent with a model where active demethylation follows recombination.

### Junctional Demethylation Is Tissue Specific

**Figure 7:**
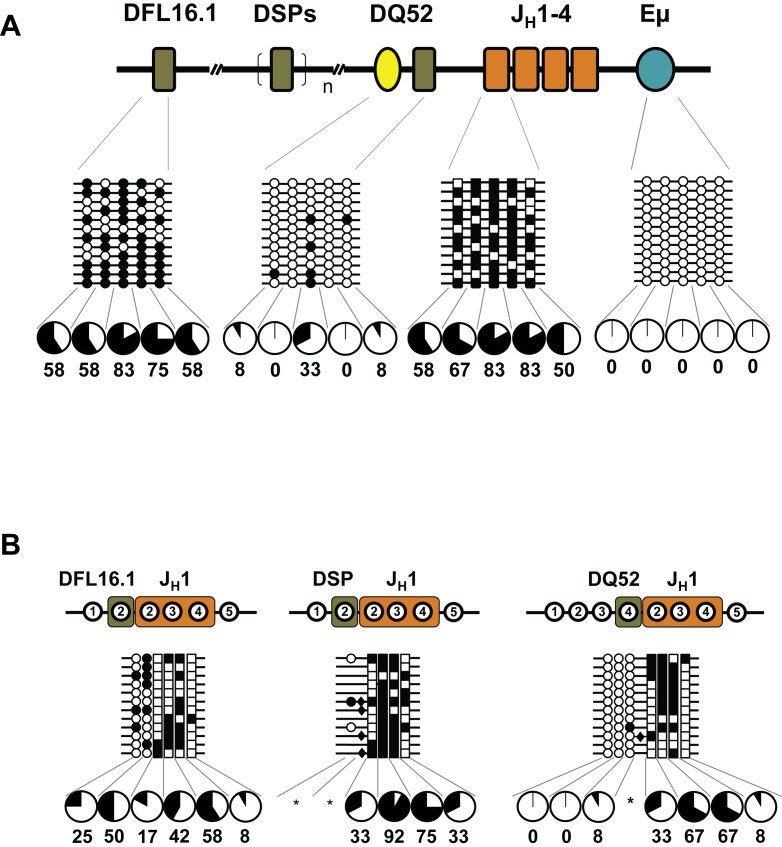
DNA methylation of IgH alleles in thymocytes. CD4^+^CD8^+^ thymocytes from C57BL/6 mice were enriched by adsorption to PNA-coated plastic plates. Genomic DNA purified from these cells was used in bisulfite modification analysis. Amplicons corresponding to unrearranged parts of IgH allele (A) and DJ_H_ junctions (B) were cloned and sequenced. Circles and squares depict CpGs corresponding to D_H_ and J_H_1 cytosines, respectively. Filled circles/squares correspond to methylated cytosines; pie charts summarize the percentage of methylated cytosines at each position except where the number of sequenced alleles falls below 12, indicated by asterisks. Untemplated CpGs incorporated during VDJ recombination were found to be methylated (filled diamonds). Data shown were obtained from two independent preparations of thymocytes, using one mouse per experiment.

D_H_ to J_H_ recombination does not occur exclusively in B lymphocyte precursors. In particular, DJ_H_ junctions have been detected in a significant proportion of DP thymocytes [42],[43]. To determine whether DJ_H_ junction demethylation was lineage restricted, we analyzed DNA from DP thymocytes from wild-type mice. We found that DQ52 and Eμ regions were demethylated, and germline DFL16.1 and J_H_ regions were hypermethylated as seen in pro-B cells (Figure 7A). However, both DFL16.1/J_H_1 and DSP/J_H_1 junctions were considerably more methylated in DP cells than their state in pro-B cells (Figure 7B), and DQ52/J_H_1 junctions displayed the hybrid phenotype seen in Eμ-deficient pro-B cells. In these cases of methylated DJ_H_ junctions we again observed de novo methylation of non-templated N region CpGs (Figure 7B, indicated by black diamonds). Thus, despite the presence of an intact demethylated enhancer, DJ_H_ junctions in DP cells retained the methylation status of the rearranging gene segments. Our interpretation is that D_H_ to J_H_ recombination does not require prior DNA demethylation. After rearrangement, Eμ activity drives demethylation in pro-B cells; however, the milieu in DP thymocytes in terms of Eμ activity or availability of transcription factors, does not permit efficient junctional DJ_H_ demethylation.

## Discussion

Systematic analysis of DNA methylation of unrearranged and partially rearranged IgH alleles in primary cells revealed several interesting features. First, the lack of correlation between histone modifications and DNA methylation status indicates that these marks are independently regulated. This was most clearly evident in the J_H_ region that is marked with the highest levels of histone acetylation and H3K4me3 in the unrearranged state, yet remains hypermethylated. Furthermore, germline DFL16.1 and the DSP gene segments were comparably methylated despite being differentially marked at the level of histone modifications. These observations appear to be at odds with genomic studies that show a correlation between histone acetylation and DNA demethylation, and H3K9 methylation and DNA methylation [31],[44],[45]. We suggest that the results from genome-wide studies may be skewed towards promoter-based CGIs. Instead, the sites we analyzed were largely non-CGIs and included enhancers (such as Eμ), promoters (such as DQ52), cryptic promoters (such as DSP and DFL16.1, see below), and regions that cannot be categorized as any of these.

An important corollary of this observation is that pre-rearrangement DNA methylation status correlates poorly with recombination potential. Recent studies identified the J_H_ region as a RAG1/2-rich recombination center [21]. Our observation suggests that DNA demethylation is not required to generate the recombination center. Additionally, DFL16.1 and DQ52 gene segments that rearrange most frequently [32]–[35] have very different levels of DNA methylation prior to rearrangement; conversely, DSP gene segments that rearrange relatively less frequently have comparable levels of DNA methylation as DFL16.1. Taken together, our working model is that DNA methylation status does not guide the first step of IgH gene assembly.

We note, however, that we cannot unequivocally rule out the possibility that selective demethylation of specific D_H_ and J_H_ segments occur in a subset of cells just prior to recombination. For example, while the J_H_1 region that we analyzed was largely methylated in pro-B cells prior to rearrangement, we observed that a minority of alleles were demethylated in both RAG-sufficient and RAG-deficient pro-B cells. These demethylated J_H_1 alleles may represent a subset of cells in which D_H_ recombination will occur preferentially to the J_H_1 gene segment. It will be interesting to determine whether other J_H_ gene segments are similarly singled out for demethylation in subsets of pro-B cells.

Second, sites of maximal tissue-specific CpG demethylation in the germline IgH locus corresponded to the two strongest DNase 1 hypersensitive sites in the 70-kb D_H_–Cμ domain, DQ52 and Eμ. Because these regions share transcriptional regulatory properties, it is likely that their demethylation is linked to transcription or transcription-associated chromatin changes. Additionally, we noted highly selective CpG demethylation of some sequences and partial demethylation of others 5′ of DFL16.1 where three additional DNase 1 HSs have been recently identified. Though these observations link DNase 1 hypersensitivity to DNA methylation, this doesn't seem to be always the case. For example, J_H_ region is known to be highly sensitive to DNase 1 digestion [46] (without being associated with “classical” DNase 1 hypersensitive sites), yet it is not demethylated. A possible model for this pattern of methylation emerges from the recent demonstration that Eμ is in spatial proximity to the cluster of DHSs 5′ of DFL16.1 [27],[47],[48]. Since Eμ itself has a demethylating potential (Eμ flanking regions are methylated on Eμ-deficient alleles), perhaps Eμ looping to 5′ DFL16.1 sites “delivers” demethylating activities to this region. The partial demethylation observed at 5′ DFL16.1 sites may reflect that such loops are present in only a fraction of pro-B cells at any given time. Consistent with the idea that Eμ induces demethylation in a subset of cells, the form of partial demethylation seen at DFL16.1 (−6.5) involves extensive demethylation of some alleles and essentially complete methylation of others (Figure 3). Eμ has also been proposed to loop to DHS5-7 of the 3′ regulatory region at the 3′ of the IgH locus. Interestingly, earlier studies by Giambra et al. [49] showed that DHS5-7 was partially demethylated in a pro-B cell line in a manner similar to the methylation state 5′ of DFL16.1. This pattern may be the consequence of Eμ looping to DHS5-7 in a subset of pro-B cells. Overall, we propose that the pattern of DNA demethylation in the germline IgH locus is determined by spatial proximity to Eμ.

Third, we found that DJ_H_ junctions were extensively demethylated. Though we cannot discount the possibility that germline D_H_ and J_H_ gene segments were demethylated just before recombination, we favor the hypothesis that demethylation of DJ_H_ junction occurred after recombination. The reasons for this are two-fold: first, DJ_H_ junctions remain methylated in Eμ-deficient pro-B cells and in CD4^+^CD8^+^ thymocytes. Our interpretation is that these DJ_H_ alleles are caught in an intermediate stage where recombination has occurred but demethylation has not. Second, we think it is unlikely that demethylation occurred prior to recombination followed by re-methylation of DJ_H_ junctions based on the state of DQ52/J_H_1 junctions. In both Eμ-deficient pro-B cells and DP thymocytes the methylation status of DQ52/J_H_1 junctions was such that the DQ52 portion was demethylated and the J_H_1 portion was methylated. If demethylation preceded rearrangement, generation of each hybrid junction would require re-methylation of a subset of closely associated CpGs.

Though our data clearly demonstrate that DNA demethylation of associated gene segments is not essential for D_H_ to J_H_ recombination, we note that the two instances of methylated DJ_H_ junctions identified in this study involve circumstances where the frequency of D_H_ recombination is lower than in wild-type pro-B cells. D_H_ recombination has been estimated to be 5- to 10-fold lower in Eμ-deleted pro-B cells [39] and only about 30%–50% of IgH alleles in DP thymocytes [42] have DJ_H_ junctions. We cannot rule out, therefore, that gene segment demethylation may increase the efficiency of D_H_ recombination in wild-type pro-B cells. Because Eμ deletion also leads to loss of other accessibility-associated epigenetic marks in the unrearranged locus, it is difficult to deconvolute the contribution of each mark to recombination efficiency.

How might DNA demethylation be targeted to DJ_H_ junctions? We have previously shown that D_H_ recombination activates cryptic bi-directional promoters associated with most D_H_ gene segments [23]. Transcriptional activity and associated RNA polymerase II recruitment is restricted to the DJ_H_ junctions, and drops off before the unrearranged D_H_ segments located 5′ of the junction. These changes require Eμ and the simplest interpretation is that recombination places cryptic D_H_ promoters under the influence of Eμ. Our working model is that D_H_-promoter/Eμ interaction brings Eμ-associated demethylating activity to DJ_H_ junctions, thereby resulting in Eμ-directed demethylation. Because Eμ's influence does not extend to the next upstream D_H_ gene segment, DJ_H_ demethylation is highly localized. In this model the substantial demethylation of DJ_H_ junctions compared to partial demethylation of looping sites may be due to stronger interaction of Eμ with DJ_H_ promoters compared to Eμ interaction with the 3′ RR or 5′ of DFL16.1 sequences.

Does DJ_H_ demethylation serve a function? In this regard it is interesting to note that V_H_ recombination is significantly reduced in both instances where DJ_H_ junctions remain methylated (Eμ-deleted alleles and in DP thymocytes). Though this is consistent with the view that DJ_H_ demethylation facilitates V_H_ recombination, we think that the regulation of V_H_ recombination is more complex. The highly localized Eμ-dependent DJ_H_ demethylation that we describe in this report adds to the emerging evidence that DJ_H_ junctions are distinguished from un-rearranged D_H_ gene segments by several forms of epigenetic changes [41]. These include activation-associated histone modifications, such as H3 acetylation and H3K4me3 and increased sensitivity to DNase I. Like DJ_H_ demethylation, these alterations are also restricted to DJ_H_ junctions and are Eμ dependent. Taken together, our working model is that all these DJ_H_-restricted epigenetic changes work in concert to promote the timing and precision of V_H_ recombination.

## Materials and Methods

### Mice

Rag2-deficient and C57BL/6 mice were obtained from Jackson Laboratory (Bar Harbor, Maine) and housed in pathogen-free facilities at the NIA or at the University of Massachusetts Medical School. Eμ-deficient mice were generated and maintained in the facilities at Childrens Hospital (Boston, Massachusetts). Mouse experiments were approved by the Animal Care and Use Committees at the NIA, Harvard Medical School, and the University of Massachusetts Medical School.

### Cell Purification

A magnetic cell-sorting system was used to purify pro-B and mature B cells as previously described [25]. Briefly, to obtain pro-B cells with IgH in germline configuration bone marrow cells were recovered from 6–8-wk-old RAG2-deficient mice by flushing the femur and tibia with 10% calf serum in PBS. CD19+ cells were purified from single cell suspension of bone marrow by positive selection using paramagnetic microbeads (Miltenyi Biotech). To obtain mature B cells, the same procedure was followed using cells obtained from the spleen of C57BL/6 mice. The resulting cells were greater than 90% CD19+ by flow cytometry.

### FACS Sorting of Pre-B Cells and Pro-B cells from C57BL/6 Mice

Freshly isolated bone marrow cells were re-suspended to 6×10^7^/ml in staining media containing biotin-, flavin-, and phenol red-deficient RPMI 1640 (Irvine Scientific), 10 mM Hepes, pH 7.2, 0.02% sodium azide, 1 mM EDTA, and 3% newborn calf serum, and treated with Fc block for 10 min on ice. Cells were incubated with primary antibodies for 20 min and then washed three times, incubated with SA-Cy5PE for 15 min, and then washed three more times. After the final wash, samples were re-suspended in 1 µg/ml propidium iodide to exclude dead cells. Primary antibodies included B220 APC; CD43 (clone S7) PE; IgM FITC; Ly6C FITC; BP-1 (clone 6C3) biotin. Antibodies were purchased from BD Biosciences/Pharmingen, eBioscience, or CALTAG. FACS Sorting was performed on a 3-laser, 9-detector MoFlo. Data were analyzed for presentation purposes with FlowJo software (Tree Star).

### CD4^+^CD8^+^ Cells

DP thymocytes were purified by PNA agglutination. In brief, equal volumes of cell suspension (7–8×10^8^ cells/ml) and PNA solution (1.5 mg/ml) were incubated for 1 h at 4°C, followed by sedimentation in fetal calf serum (Invitrogen). PNA-agglutinated cell pellet was dissociated by D-galactose treatment resulting in greater than 90% pure CD4^+^CD8^+^ cells.

### Genomic DNA Isolation and Modification

Genomic DNA was extracted from 1–5×10^6^ cells by using the DNeasy Blood & Tissue Kit (QIAGEN) according to the manufacturer's protocol.

Sodium bisulfite modification was carried out as described earlier [28] with some modifications. Briefly, 1 µg of genomic DNA was denatured by incubating with NaOH at 37°C for 15 min. Sodium metabisulfite (3 M) and hydriquinone (0.5 mM) were added to the samples together and incubated overnight at 55°C. The reaction was stopped by incubating samples with NaOH 15 min at 37°C. Modified DNA was purified using GeneClean II (MP Biomedicals). Modified DNA was precipitated and dissolved in 30 µl of 1 mM Tris-HCl (pH 8.0).

Modified templates were amplified by nested PCR using primers listed in Table S2. PCR products were separated by agarose gel electrophoresis, purified using QIAquick gel extraction kit (QIAGEN), and cloned into pCRII-TOPO vector. Mini prep DNA containing amplicon inserts were identified by PCR and sequenced commercially (SeqWright). Sequence analysis showed 99%–100% bisulfite modification efficiency (Figure S6).

## Supporting Information

Figure S1 **Comparison of DNA methylation status between two independent experiments.** For each region analyzed in the paper, we used at least two independent DNA preparations starting with cells obtained from six mice for each sample. Methylation profiles of the regions amplified from two independent experiments were comparable, as shown with the example of the DFL16.1 region in RAG^−/−^ pro-B mice.
(TIF)

Figure S2 **DNA methylation state of the mb-1 and β-globin locus activating region in different cell types.** For our experiments we have chosen the previously characterized mb-1 gene and its promoter as a positive and β-globin locus activating region as a negative control. Sequences from at least 12 individual colonies were analyzed for each cell type. Consistent with the observations of the Hagman group [29] mb-1 gene and its promoter are hypomethylated in early stages of B cell development. As expected mb-1 and its promoter were methylated in CD4^+^CD8^+^ DP thymocytes and kidney cells, which served as a negative control for this region. β-globin locus activating region was methylated in a RAG2-deficient pro-B cell line, primary RAG2-deficient pro-B cells, and kidney.
(TIF)

Figure S3 **Relative positions of the CpG dinucleotides in the amplicons analyzed.** Amplicons covering 11 regions of the germline IgH locus are depicted. Numbers in circles represent the order of the CpGs in each amplicon. D_H_ gene segments are highlighted in green, J_H_1 region is highlighted in orange. Recombination signal sequences (RSSs) with 12- and 23-bp spacers are highlighted in light blue and grey, respectively. Distances (in nucleotides) between CpG dinucleotides and gene segments are indicated.
(TIF)

Figure S4 **Analysis of individual sequences at DJ_H_ junctions.** While analyzing DNA methylation profiles, one question arises especially in the case of demethylated sequences: whether this is a single DNA molecule having been amplified during PCR. To answer that question we have performed a careful analysis of DNA at the joining regions; nucleotides introduced by non-templated end-joining permit unequivocal assignment of sequences to individual alleles. Aligned sequences of a representative cell type, pre-B cells, are shown here.
(TIF)

Figure S5 **DNA methylation state of the Eμ region in Eμ^−^ pro-B cells.** The schematic representation of the Eμ region in Eμ^−^ cells is represented on top. CpGs are depicted as vertical bars; those CpGs that correspond to the ones in the wild-type sequence adjacent to the Eμ deletion sites, are depicted in blue color, the ones that are introduced during cre-deletion in brown.
(TIF)

Figure S6 **Bisulfite modification efficiency.** We compared each modified sequence with the genomic DNA sequence to determine the efficiency of cytosine conversion. Sequences used in the analysis showed 99%–100% modification efficiency.
(TIF)

Table S1 **Summary of bisulfite modification analysis.** Number of sequences analyzed for each amplicon (gene segment) in each cell type (cell type) are indicated (number of sequences).
(DOC)

Table S2 **Primer sequences used to amplify indicated amplicons for bisulfite modification analysis.**
(DOC)

## Abbreviations

CGI: CpG islands
D_H_: IgH diversity region
DHS: DNase l hypersensitive sites
DP: double positive
H3K4me3: histone H3 trimethylated at lysine 4
H3K9me: histone H3 methylated at lysine 9
H3K9me2: H3 dimethylated at lysine 9
IgH: immunoglobulin heavy chain
J_H_: IgH joining region
V_H_: IgH variable region

## References

[1] Bird A (2002) DNA methylation patterns and epigenetic memory. Genes Dev 16: 6–21. 11782440 10.1101/gad.947102

[2] Kouzarides T (2007) Chromatin modifications and their function. Cell 128: 693–705. 17320507 10.1016/j.cell.2007.02.005

[3] Schneider R, Grosschedl R (2007) Dynamics and interplay of nuclear architecture, genome organization, and gene expression. Genes Dev 21: 3027–3043. 18056419 10.1101/gad.1604607

[4] Sexton T, Schober H, Fraser P, Gasser SM (2007) Gene regulation through nuclear organization. Nat Struct Mol Biol 14: 1049–1055. 17984967 10.1038/nsmb1324

[5] Lee TI, Jenner RG, Boyer LA, Guenther MG, Levine SS, et al (2006) Control of developmental regulators by Polycomb in human embryonic stem cells. Cell 125: 301–313. 16630818 10.1016/j.cell.2006.02.043 PMC3773330

[6] Vaissiere T, Sawan C, Herceg Z (2008) Epigenetic interplay between histone modifications and DNA methylation in gene silencing. Mutat Res 659: 40–48. 18407786 10.1016/j.mrrev.2008.02.004

[7] Jones PA (2012) Functions of DNA methylation: islands, start sites, gene bodies and beyond. Nat Rev Genet 13: 484–492. 22641018 10.1038/nrg3230

[8] Maunakea AK, Nagarajan RP, Bilenky M, Ballinger TJ, D'Souza C, et al (2010) Conserved role of intragenic DNA methylation in regulating alternative promoters. Nature 466: 253–257. 20613842 10.1038/nature09165 PMC3998662

[9] Deaton AM, Webb S, Kerr AR, Illingworth RS, Guy J, et al (2011) Cell type-specific DNA methylation at intragenic CpG islands in the immune system. Genome Res 21: 1074–1086. 21628449 10.1101/gr.118703.110 PMC3129250

[10] Bassing CH, Swat W, Alt FW (2002) The mechanism and regulation of chromosomal V(D)J recombination. Cell 109 Suppl: S45–55. 11983152 10.1016/s0092-8674(02)00675-x

[11] Chevillard C, Ozaki J, Herring CD, Riblet R (2002) A three-megabase yeast artificial chromosome contig spanning the C57BL mouse Igh locus. J Immunol 168: 5659–5666. 12023364 10.4049/jimmunol.168.11.5659

[12] Johnston CM, Wood AL, Bolland DJ, Corcoran AE (2006) Complete sequence assembly and characterization of the C57BL/6 mouse Ig heavy chain V region. J Immunol 176: 4221–4234. 16547259 10.4049/jimmunol.176.7.4221

[13] Mather EL, Perry RP (1983) Methylation status and DNase I sensitivity of immunoglobulin genes: changes associated with rearrangement. Proc Natl Acad Sci U S A 80: 4689–4693. 6308642 10.1073/pnas.80.15.4689 PMC384109

[14] Storb U, Arp B (1983) Methylation patterns of immunoglobulin genes in lymphoid cells: correlation of expression and differentiation with undermethylation. Proc Natl Acad Sci U S A 80: 6642–6646. 6314334 10.1073/pnas.80.21.6642 PMC391226

[15] Mostoslavsky R, Singh N, Kirillov A, Pelanda R, Cedar H, et al (1998) Kappa chain monoallelic demethylation and the establishment of allelic exclusion. Genes Dev 12: 1801–1811. 9637682 10.1101/gad.12.12.1801 PMC316908

[16] Engler P, Storb U (1999) Hypomethylation is necessary but not sufficient for V(D)J recombination within a transgenic substrate. Mol Immunol 36: 1169–1173. 10698319 10.1016/s0161-5890(99)00124-8

[17] Engler P, Weng A, Storb U (1993) Influence of CpG methylation and target spacing on V(D)J recombination in a transgenic substrate. Mol Cell Biol 13: 571–577. 8417353 10.1128/mcb.13.1.571-577.1993 PMC358936

[18] Fraenkel S, Mostoslavsky R, Novobrantseva TI, Pelanda R, Chaudhuri J, et al (2007) Allelic ‘choice’ governs somatic hypermutation in vivo at the immunoglobulin kappa-chain locus. Nat Immunol 8: 715–722. 17546032 10.1038/ni1476

[19] Johnson K, Pflugh DL, Yu D, Hesslein DG, Lin KI, et al (2004) B cell-specific loss of histone 3 lysine 9 methylation in the V(H) locus depends on Pax5. Nat Immunol 5: 853–861. 15258579 10.1038/ni1099 PMC1635547

[20] Espinoza CR, Feeney AJ (2007) Chromatin accessibility and epigenetic modifications differ between frequently and infrequently rearranging VH genes. Mol Immunol 44: 2675–2685. 17218014 10.1016/j.molimm.2006.12.002 PMC2570232

[21] Ji Y, Resch W, Corbett E, Yamane A, Casellas R, et al (2010) The in vivo pattern of binding of RAG1 and RAG2 to antigen receptor loci. Cell 141: 419–431. 20398922 10.1016/j.cell.2010.03.010 PMC2879619

[22] Featherstone K, Wood AL, Bowen AJ, Corcoran AE (2011) The mouse immunoglobulin heavy chain V-D intergenic sequence contains insulators that may regulate ordered V(D)J recombination. J Biol Chem 285: 9327–9338. 10.1074/jbc.M109.098251 PMC2843181 20100833

[23] Chakraborty T, Chowdhury D, Keyes A, Jani A, Subrahmanyam R, et al (2007) Repeat organization and epigenetic regulation of the DH-Cmu domain of the immunoglobulin heavy-chain gene locus. Mol Cell 27: 842–850. 17803947 10.1016/j.molcel.2007.07.010

[24] Morshead KB, Ciccone DN, Taverna SD, Allis CD, Oettinger MA (2003) Antigen receptor loci poised for V(D)J rearrangement are broadly associated with BRG1 and flanked by peaks of histone H3 dimethylated at lysine 4. Proc Natl Acad Sci U S A 100: 11577–11582. 14500909 10.1073/pnas.1932643100 PMC208800

[25] Chowdhury D, Sen R (2001) Stepwise activation of the immunoglobulin mu heavy chain gene locus. Embo J 20: 6394–6403. 11707410 10.1093/emboj/20.22.6394 PMC125735

[26] Degner SC, Wong TP, Jankevicius G, Feeney AJ (2009) Cutting edge: developmental stage-specific recruitment of cohesin to CTCF sites throughout immunoglobulin loci during B lymphocyte development. J Immunol 182: 44–48. 19109133 10.4049/jimmunol.182.1.44 PMC2625297

[27] Guo C, Yoon HS, Franklin A, Jain S, Ebert A, et al (2011) CTCF-binding elements mediate control of V(D)J recombination. Nature 477: 424–430. 21909113 10.1038/nature10495 PMC3342812

[28] Frommer M, McDonald LE, Millar DS, Collis CM, Watt F, et al (1992) A genomic sequencing protocol that yields a positive display of 5-methylcytosine residues in individual DNA strands. Proc Natl Acad Sci U S A 89: 1827–1831. 1542678 10.1073/pnas.89.5.1827 PMC48546

[29] Maier H, Ostraat R, Gao H, Fields S, Shinton SA, et al (2004) Early B cell factor cooperates with Runx1 and mediates epigenetic changes associated with mb-1 transcription. Nat Immunol 5: 1069–1077. 15361869 10.1038/ni1119

[30] Kiefer CM, Hou C, Little JA, Dean A (2008) Epigenetics of beta-globin gene regulation. Mutat Res 647: 68–76. 18760288 10.1016/j.mrfmmm.2008.07.014 PMC2617773

[31] Esteve PO, Chin HG, Smallwood A, Feehery GR, Gangisetty O, et al (2006) Direct interaction between DNMT1 and G9a coordinates DNA and histone methylation during replication. Genes Dev 20: 3089–3103. 17085482 10.1101/gad.1463706 PMC1635145

[32] Bangs LA, Sanz IE, Teale JM (1991) Comparison of D, JH, and junctional diversity in the fetal, adult, and aged B cell repertoires. J Immunol 146: 1996–2004. 1672338

[33] Chang Y, Paige CJ, Wu GE (1992) Enumeration and characterization of DJH structures in mouse fetal liver. Embo J 11: 1891–1899. 1582417 10.1002/j.1460-2075.1992.tb05241.x PMC556647

[34] Feeney AJ (1990) Lack of N regions in fetal and neonatal mouse immunoglobulin V-D-J junctional sequences. J Exp Med 172: 1377–1390. 1700054 10.1084/jem.172.5.1377 PMC2188672

[35] Gu H, Kitamura D, Rajewsky K (1991) B cell development regulated by gene rearrangement: arrest of maturation by membrane-bound D mu protein and selection of DH element reading frames. Cell 65: 47–54. 2013094 10.1016/0092-8674(91)90406-o

[36] Bolland DJ, Wood AL, Afshar R, Featherstone K, Oltz EM, et al (2007) Antisense intergenic transcription precedes Igh D-to-J recombination and is controlled by the intronic enhancer Emu. Mol Cell Biol 27: 5523–5533. 17526723 10.1128/MCB.02407-06 PMC1952079

[37] Jung D, Giallourakis C, Mostoslavsky R, Alt FW (2006) Mechanism and control of V(D)J recombination at the immunoglobulin heavy chain locus. Annu Rev Immunol 24: 541–570. 16551259 10.1146/annurev.immunol.23.021704.115830

[38] Dudley DD, Sekiguchi J, Zhu C, Sadofsky MJ, Whitlow S, et al (2003) Impaired V(D)J recombination and lymphocyte development in core RAG1-expressing mice. J Exp Med 198: 1439–1450. 14581608 10.1084/jem.20030627 PMC2194253

[39] Afshar R, Pierce S, Bolland DJ, Corcoran A, Oltz EM (2006) Regulation of IgH gene assembly: role of the intronic enhancer and 5′DQ52 region in targeting DHJH recombination. J Immunol 176: 2439–2447. 16456003 10.4049/jimmunol.176.4.2439

[40] Perlot T, Alt FW, Bassing CH, Suh H, Pinaud E (2005) Elucidation of IgH intronic enhancer functions via germ-line deletion. Proc Natl Acad Sci U S A 102: 14362–14367. 16186486 10.1073/pnas.0507090102 PMC1242331

[41] Subrahmanyam R, Du H, Ivanova I, Chakraborty T, Ji Y, et al (2012) Localized epigenetic changes induced by D(H) recombination restricts recombinase to DJ(H) junctions. Nat Immunol 10.1038/ni.2447 PMC3685187 23104096

[42] Subrahmanyam R, Du H, Ivanova I, Chakraborty T, Ji Y, et al (2012) Localized epigenetic changes induced by D(H) recombination restricts recombinase to DJ(H) junctions. Nat Immunol 13: 1205–1212. 23104096 10.1038/ni.2447 PMC3685187

[43] Hsu LY, Liang HE, Johnson K, Kang C, Schlissel MS (2004) Pax5 activates immunoglobulin heavy chain V to DJ rearrangement in transgenic thymocytes. J Exp Med 199: 825–830. 15007090 10.1084/jem.20032249 PMC2212727

[44] Jones PL, Veenstra GJ, Wade PA, Vermaak D, Kass SU, et al (1998) Methylated DNA and MeCP2 recruit histone deacetylase to repress transcription. Nat Genet 19: 187–191. 9620779 10.1038/561

[45] Stancheva I (2005) Caught in conspiracy: cooperation between DNA methylation and histone H3K9 methylation in the establishment and maintenance of heterochromatin. Biochem Cell Biol 83: 385–395. 15959564 10.1139/o05-043

[46] Maes J, Chappaz S, Cavelier P, O'Neill L, Turner B, et al (2006) Activation of V(D)J recombination at the IgH chain JH locus occurs within a 6-kilobase chromatin domain and is associated with nucleosomal remodeling. J Immunol 176: 5409–5417. 16622008 10.4049/jimmunol.176.9.5409

[47] Guo C, Gerasimova T, Hao H, Ivanova I, Chakraborty T, et al (2011) Two forms of loops generate the chromatin conformation of the immunoglobulin heavy-chain gene locus. Cell 147: 332–343. 21982154 10.1016/j.cell.2011.08.049 PMC3685183

[48] Degner SC, Verma-Gaur J, Wong TP, Bossen C, Iverson GM, et al (2011) CCCTC-binding factor (CTCF) and cohesin influence the genomic architecture of the Igh locus and antisense transcription in pro-B cells. Proc Natl Acad Sci U S A 108: 9566–9571. 21606361 10.1073/pnas.1019391108 PMC3111298

[49] Giambra V, Volpi S, Emelyanov AV, Pflugh D, Bothwell AL, et al (2008) Pax5 and linker histone H1 coordinate DNA methylation and histone modifications in the 3′ regulatory region of the immunoglobulin heavy chain locus. Mol Cell Biol 28: 6123–6133. 18644860 10.1128/MCB.00233-08 PMC2547000

