# Supplementary figures and images for: Localized DNA Demethylation at Recombination Intermediates during Immunoglobulin Heavy Chain Gene Assembly
Source: PLoS Biol. 2013 Jan 29;11(1):e1001475. doi: 10.1371/journal.pbio.1001475 (PMC3558432; doi:10.1371/journal.pbio.1001475)

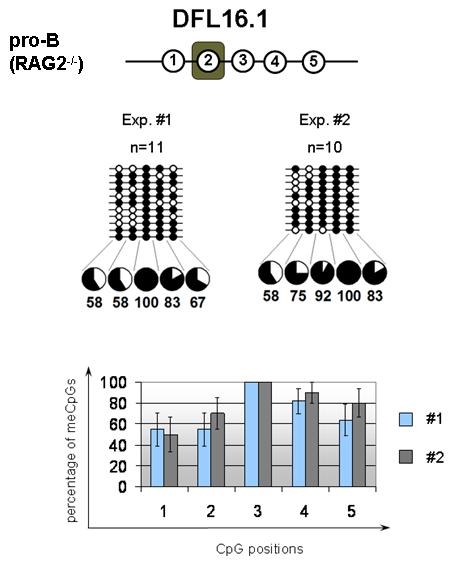

Supplement: Figure S1 — Comparison of DNA methylation status between two independent experiments. For each region analyzed in the paper, we used at least two independent DNA preparations starting with cells obtained from six mice for each sample. Methylation profiles of the regions amplified from two independent experiments were comparable, as shown with the example of the DFL16.1 region in RAG−/− pro-B mice. (TIF) [file pbio.1001475.s001.tif]

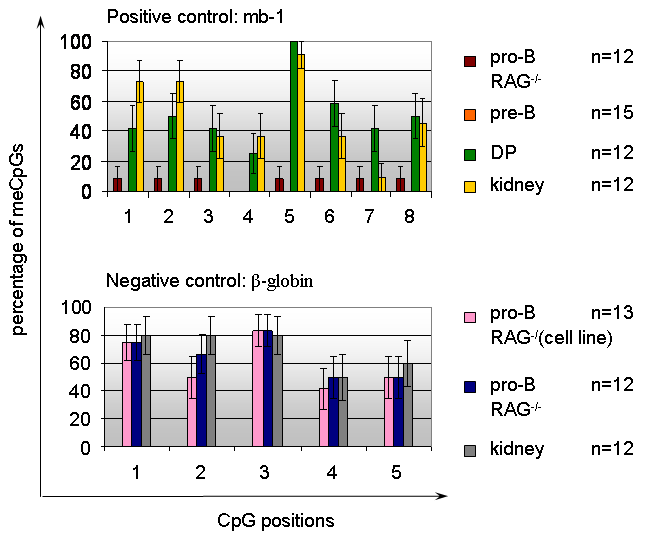

Supplement: Figure S2 — DNA methylation state of the mb-1 and β-globin locus activating region in different cell types. For our experiments we have chosen the previously characterized mb-1 gene and its promoter as a positive and β-globin locus activating region as a negative control. Sequences from at least 12 individual colonies were analyzed for each cell type. Consistent with the observations of the Hagman group [29] mb-1 gene and its promoter are hypomethylated in early stages of B cell development. As expected mb-1 and its promoter were methylated in CD4+CD8+ DP thymocytes and kidney cells, which served as a negative control for this region. β-globin locus activating region was methylated in a RAG2-deficient pro-B cell line, primary RAG2-deficient pro-B cells, and kidney. (TIF) [file pbio.1001475.s002.tif]

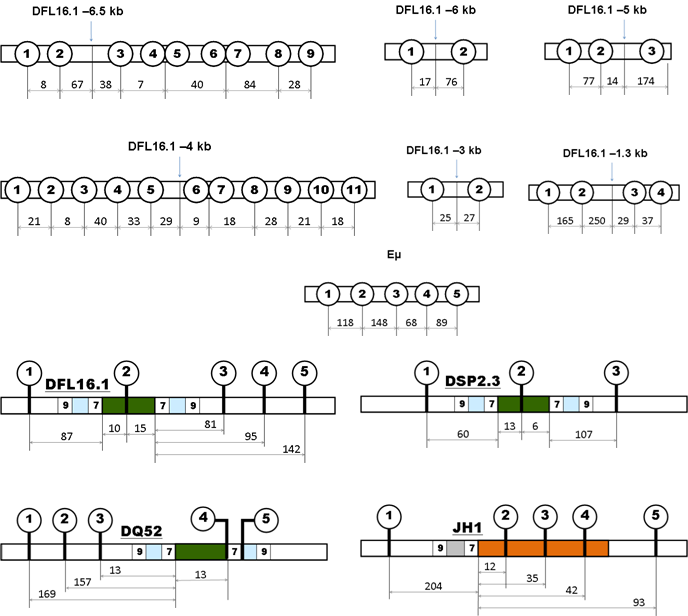

Supplement: Figure S3 — Relative positions of the CpG dinucleotides in the amplicons analyzed. Amplicons covering 11 regions of the germline IgH locus are depicted. Numbers in circles represent the order of the CpGs in each amplicon. DH gene segments are highlighted in green, JH1 region is highlighted in orange. Recombination signal sequences (RSSs) with 12- and 23-bp spacers are highlighted in light blue and grey, respectively. Distances (in nucleotides) between CpG dinucleotides and gene segments are indicated. (TIF) [file pbio.1001475.s003.tif]

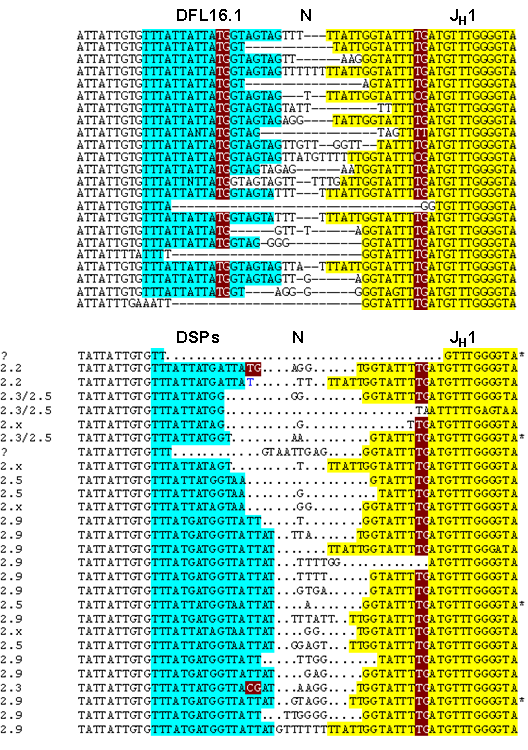

Supplement: Figure S4 — Analysis of individual sequences at DJH junctions. While analyzing DNA methylation profiles, one question arises especially in the case of demethylated sequences: whether this is a single DNA molecule having been amplified during PCR. To answer that question we have performed a careful analysis of DNA at the joining regions; nucleotides introduced by non-templated end-joining permit unequivocal assignment of sequences to individual alleles. Aligned sequences of a representative cell type, pre-B cells, are shown here. (TIF) [file pbio.1001475.s004.tif]

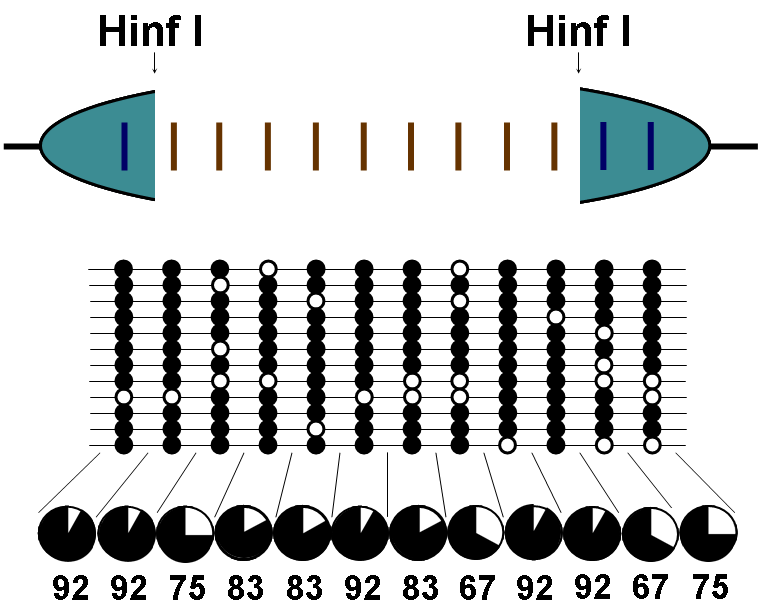

Supplement: Figure S5 — DNA methylation state of the Eμ region in Eμ− pro-B cells. The schematic representation of the Eμ region in Eμ− cells is represented on top. CpGs are depicted as vertical bars; those CpGs that correspond to the ones in the wild-type sequence adjacent to the Eμ deletion sites, are depicted in blue color, the ones that are introduced during cre-deletion in brown. (TIF) [file pbio.1001475.s005.tif]

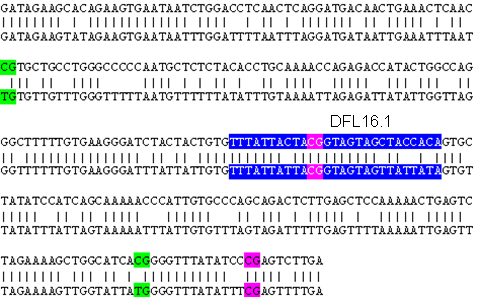

Supplement: Figure S6 — Bisulfite modification efficiency. We compared each modified sequence with the genomic DNA sequence to determine the efficiency of cytosine conversion. Sequences used in the analysis showed 99%–100% modification efficiency. (TIF) [file pbio.1001475.s006.tif]
