# Supplementary material for: Localized DNA Demethylation at Recombination Intermediates during Immunoglobulin Heavy Chain Gene Assembly
Source: PLoS Biol. 2013 Jan 29;11(1):e1001475. doi: 10.1371/journal.pbio.1001475 (PMC3558432; doi:10.1371/journal.pbio.1001475)
Supplement: Table S1 — Summary of bisulfite modification analysis. Number of sequences analyzed for each amplicon (gene segment) in each cell type (cell type) are indicated (number of sequences). (DOC) [file pbio.1001475.s007.doc]

**Supplementary Table 1**

| **Gene segment** | **Cell type** | **Number of sequences** |
| --- | --- | --- |
| DFL16 | RAG2^-/-^ pro-B | 22 |
|  | pro-B | 36 |
|  | pre-B | 15 |
|  | mature B | 15 |
|  | CD4^+^CD8^+^ | 19 |
|  | Eμ^-/-^ pro-B | 21 |
|  | RAG-/- cell line | 50 |
|  | kidney | 15 |
| DSPs | RAG2^-/-^ pro-B | 42 |
|  | pro-B | 16 |
|  | pre-B | 22 |
|  | RAG^-/-^ cell line | 15 |
|  | kidney | 15 |
| DQ52 | RAG2^-/-^ pro-B | 12 |
|  | Eμ^-/-^ pro-B | 13 |
|  | CD4^+^CD8^+^ | 16 |
|  | kidney | 13 |
| J_H_1 | RAG2^-/-^ pro-B | 13 |
|  | pro-B | 25 |
|  | pre-B | 15 |
|  | CD4^+^CD8^+^ | 14 |
|  | Eμ^-/-^ pro-B | 16 |
|  | kidney | 16 |
| DFL16-JH1 | pro-B | 29 |
|  | pre-B | 34 |
|  | mature B | 15 |
|  | CD4^+^CD8^+^ | 17 |
|  | Eμ^-/-^ pro-B | 13 |
| DSPs-JH1 | pro-B | 43 |
|  | pre-B | 16 |
|  | mature B | 32 |
|  | CD4^+^CD8^+^ | 38 |
|  | Eμ^-/-^ pro-B | 34 |
| DQ52-JH1 | Pre-B | 28 |
|  | mature B | 21 |
|  | CD4^+^CD8^+^ | 31 |
|  | Eμ^-/-^ pro-B | 12 |
| Eμ | RAG2^-/-^ pro-B | 14 |
|  | pro-B | 13 |
|  | pre-B | 13 |
|  | mature B | 15 |
|  | CD4^+^CD8^+^ | 21 |
|  | Eμ^-/-^ pro-B | 15 |
|  | kidney | 15 |
| mb-1 | RAG2^-/-^ pro-B | 12 |
|  | pre-B | 18 |
|  | CD4^+^CD8^+^ | 14 |
|  | Eμ^-/-^ pro-B | 12 |
|  | kidney | 12 |
| DFL16 -1.3kb | RAG2^-/-^ pro-B | 15 |
|  | pro-B | 12 |
|  | mature B | 13 |
|  | Eμ^-/-^ pro-B | 16 |
| DFL16 -3kb | RAG2^-/-^ pro-B | 12 |
|  | CD4^+^CD8^+^ | 12 |
| DFL16 -4kb | pre-pro-B | 12 |
|  | CD4^+^CD8^+^ | 12 |
| DFL16 -5kb | RAG2^-/-^ pro-B | 15 |
|  | CD4^+^CD8^+^ | 12 |
| DFL16 -6kb | pre-pro-B | 12 |
|  | CD4^+^CD8^+^ | 12 |
| DFL16 -6.5kb | RAG2^-/-^ pro-B | 12 |
|  | CD4^+^CD8^+^ | 12 |

Summary of bisulfite modification analysis. Number of sequences analyzed for each amplicon (Gene Segment) in each cell type (Cell Type) are indicated (Number of sequences)
