# Supplementary material for: Localized DNA Demethylation at Recombination Intermediates during Immunoglobulin Heavy Chain Gene Assembly
Source: PLoS Biol. 2013 Jan 29;11(1):e1001475. doi: 10.1371/journal.pbio.1001475 (PMC3558432; doi:10.1371/journal.pbio.1001475)
Supplement: Table S2 — Primer sequences used to amplify indicated amplicons for bisulfite modification analysis. (DOC) [file pbio.1001475.s008.doc]

**Supplementary Table 2**

-6.5DFL16 F 5’ TTG TTA TTT TTT TAA TAT TAT TAG ATT TGA G 3’

-6.5DFL16 FN 5’ AAT TTT ATT TGG GTA TTT GTG AAT TTT AG 3’

-6.5DFL16 R 5’ TTT TTC TTC TAT CTT ATA AAT ACA TAT TAC 3’

-6.5DFL16 RN 5’ CCT ATC TTT AAA ACT ATA AAC CTT ACT C 3’

-6DFL16 F 5’ ATT ATT ATT ATT AAT TTA AGG TAT TTT ATT AG 3’

-6DFL16 FN 5’ ATT AGA TAT TTT TTT TAT TTA GTT TTT AAA TG 3’

-6DFL16 R 5’ TAC CCA AAA AAC TAA AAA AAT CTA CAA C 3’

-6DFL16 RN 5’ CTC CCC AAA ACT ATA ACT CTA ATT AC 3’

-5DFL16 F 5’ GAT TTA GAA AAT TGA TGT TAT ATA GTT AAG 3’

-5DFL16 FN 5’ ATA TAG TTA AGT GGA TTT TTA GGA ATA G 3’

-5DFL16 R 5’ ACT ATA AAA CAA ACA AAA CCA AAA TCA C 3’

-5DFL16 RN 5’ ATC ACT AAA AAA ACA TCA AAT ATA AAA AAC 3’

-4DFL16 F 5’ TAA ATT AAA GTA ATA TGA AGT TTT GTT TTG 3’

-4DFL16 FN 5’ TAG TTG TTA AAA TTT AAT TGT GTA ATA TAG 3’

-4DFL16 R 5’ CTA ATA AAA ATT AAT ACT AAA AAA TTA TCA C 3’

-4DFL16 RN 5’ TTA ATA CTA AAA AAT TAT CAC AAT TAA CAC 3’

-3DFL16 F 5’ GGA TTA TAG GAG TTG TTT TGT TTA TTA G 3’

-3DFL16 FN 5’ GGA GTT GTT TTG TTT ATT AGT TTA TTT TG 3’

-3DFL16 R 5’ CCA TAT TTC TAC CTT TCT ATA AAT TAA C 3’

-3DFL16 RN 5’ CTA TAT TCC AAA AAT AAA ATA CAA AAT ATC 3’

-1.3DFL16 F 5’ TAG TTA GTT TTA GTT TAT TTT ATG TTT TAG 3’

-1.3DFL16 FN 5’ AGA AAT AAT AGT ATT TTT ATT GTA GAT G 3’

-1.3DFL16 R 5’ ATT CCT ACT CCA AAA TAA TAA ATA TAA AC 3’

-1.3DFL16 RN 5’ CAA AAT AAT AAA TAT AAA CTA AAA ATT CAT C 3’

DFL16 F 5’ GTT AGA TTT TTT TTG TTT TAG GAT TTT TTT GAA 3’

DFL16 FN 5’ GAT AGA AGT ATA GAA GTG AAT AAT TTG G 3’

DFL16 R 5’ CTA AAA AAA AAA TCT CTA AAA CCC TTC C 3’

DFL16 RN 5’ AAC TCT AAA TAA AAA ACT TTA AAA TCT AAC 3’

DSPs F 5’ TAA GGA TGG TTT TTG ATA TTT TGT ATT G 3’

DSPs FN 5’ GGT TTT TGA TAT TTT GTA TTG TTA TTT TTG 3’

DSPs R 5’ ATA TCT AAA ATA CTA CTA TCT TAT AAA TAC 3’

DQ52 F 5’ TTT TTT TGT TGT AGA GGT GGG ATT AG 3’

DQ52 FN 5’ ATA ATT TAG ATA TAA GTG AAT GAT AGA TG 3’

DQ52 R 5’ TTT CTT CCT CAA ATT TCT CAA ACC TC 3’

DQ52 RN 5’ TCT ACT TCC TCA TAA CTC AAA CTA C 3’

JH1 F 5’ ATA GAG TAG GTA GGT GGA GTT GAT TGA G 3’

JH1 FN 5’ GAG ATT TTT TTA AAT ATT TGA GTT TTT GAG 3’

JH1 R 5’ TCC TTA CAA AAA AAC TTC TAC AAC ATA C 3’

JH1 RN 5’ CAA CAT ACA AAT ATA ACA AAT AAC CTA AC 3’

Eμ F 5’ GTT TGA GGT TTT GTT TGT GTA GAA TTG 3’

Eμ FN 5’ TTT GAG GTT TTG TTT GTG TAG AAT TGA T 3’

Eμ R 5’ ACT AAA ATC CTT CAA TTT CTT ACA TAA C 3’

Eμ RN 5’ CTT ACA TAA CCT AAT TTT AAA AAT AAA TTC 3’

β-globin F 5’ GAG TAA AGG GTT TAA TAA GAA AAA TAT AG 3’

β-globin R 5’ TAA CTC ACA AAA CTA AAC ACA CCC AC 3’

Primer sequences used to amplify indicated amplicons for bisulfite modification analysis.
